# Routes of transmission of VIM-positive *Pseudomonas aeruginosa* in the adult intensive care unit-analysis of 9 years of surveillance at a university hospital using a mathematical model

**DOI:** 10.1186/s13756-022-01095-x

**Published:** 2022-04-04

**Authors:** Thi Mui Pham, Andrea C. Büchler, Anne F. Voor in ‘t holt, Juliëtte A. Severin, Martin C. J. Bootsma, Diederik Gommers, Mirjam E. Kretzschmar, Margreet C. Vos

**Affiliations:** 1 Julius Center for Health Sciences and Primary Care, University Medical Center Utrecht, Utrecht University, Utrecht, The Netherlands; 2 Department of Medical Microbiology and Infectious Diseases, Erasmus MC University Medical Center, P.O. Box 2040, 3000 CA Rotterdam, The Netherlands; 3 Department of Mathematics, Faculty of Science, Utrecht University, Utrecht, The Netherlands; 4 Department of Adult Intensive Care, Erasmus MC University Medical Center, Rotterdam, The Netherlands

**Keywords:** Drug Resistance, multiple, Pseudomonas aeruginosa, Critical care, Epidemiological monitoring, Models, statistical

## Abstract

**Background:**

Hospital outbreaks of multidrug resistant *Pseudomonas aeruginosa* are often caused by *Pseudomonas aeruginosa* clones which produce metallo-β-lactamases, such as Verona Integron-encoded Metallo-β-lactamase (VIM). Although different sources have been identified, the exact transmission routes often remain unknown. However, quantifying the role of different transmission routes of VIM-PA is important for tailoring infection prevention and control measures. The aim of this study is to quantify the relative importance of different transmission routes by applying a mathematical transmission model using admission and discharge dates as well as surveillance culture data of patients.

**Methods:**

We analyzed VIM-PA surveillance data collected between 2010 and 2018 of two intensive-care unit (ICU) wards for adult patients of the Erasmus University Medical Center Rotterdam using a mathematical transmission model. We distinguished two transmission routes: direct cross-transmission and a persistent environmental route. Based on admission, discharge dates, and surveillance cultures, we estimated the proportion of transmissions assigned to each of the routes.

**Results:**

Our study shows that only 13.7% (95% CI 1.4%, 29%) of the transmissions that occurred in these two ICU wards were likely caused by cross-transmission, leaving the vast majority of transmissions (86.3%, 95% CI 71%, 98.6%) due to persistent environmental contamination.

**Conclusions:**

Our results emphasize that persistent contamination of the environment may be an important driver of nosocomial transmissions of VIM-PA in ICUs. To minimize the transmission risk from the environment, potential reservoirs should be regularly and thoroughly cleaned and disinfected, or redesigned.

**Supplementary Information:**

The online version contains supplementary material available at 10.1186/s13756-022-01095-x.

## Introduction

Multidrug resistant (MDR) microorganisms are an emerging problem worldwide. The most emerging threat is the spread of carbapenem-resistant Enterobacterales and carbapenem-resistant non-fermenting microorganisms, such as *Acinetobacter baumannii* and *Pseudomonas aeruginosa* [1]. *P. aeruginosa* is one of the most common nosocomial pathogens [2, 3]. It can cause serious infections in patients with underlying conditions, such as immunosuppression, cystic fibrosis, and patients admitted to the intensive care unit (ICU). The morbidity and mortality of *P. aeruginosa* bloodstream infections is high, especially in immunocompromised patients [4–6]. Due to its intrinsic and acquired resistance to multiple antibiotics, *P. aeruginosa* is not only a common cause of nosocomial infections but also difficult to treat. Multidrug resistance mechanisms in *P. aeruginosa* are loss or alteration of outer membrane porins, increased efflux pump activity and carbapenemase production [2] with the latter being the most common underlying mechanism of MDR *P. aeruginosa* involved in in-hospital outbreaks [7]. Among the carbapenemases, the Verona Integron-encoded Metallo-beta-lactamase (VIM) is most dominant, and most widely disseminated [8].

Identifying the pathways of transmission of *P. aeruginosa* in hospital outbreaks is key for targeted and timely infection prevention and control (IPC) measures. Although the exact transmission route often remains unknown, different modes of transmission are described in the literature. For *P. aeruginosa*, water-related devices such as sinks are the most common environmental source [9, 10]. Quantifying the relative importance of transmission routes may serve as an essential tool in outbreak investigation as well as in designing effective and tailored IPC strategies.

Models for inference of transmission parameters for different transmission routes have been developed for various MDR bacteria [11–14]. Pham et al. [14] developed a mathematical transmission model including three different routes of transmission for *P. aeruginosa* using ICU data from two ICUs of a French hospital in Besançon. The authors estimated the relative contribution of background transmission, cross-transmission and environmental contamination after discharge using an extensive surveillance data set. It was shown that environmental contamination due to colonized patients that persisted after their discharge likely had a small contribution (< 1%) to the overall number of transmissions. Persistent environmental contamination was included in “background transmission” for which the relative contribution was significantly higher. While this route could have played an important role in the transmission process, it could not be distinguished from other routes that could have caused a similar constant risk of colonization.

In this paper, we present the application of a similar mathematical transmission model to surveillance data of VIM-producing *P. aeruginosa* (VIM-PA) at the Erasmus University Medical Center Rotterdam (Erasmus MC), the Netherlands. In this hospital, since 2003, VIM-PA colonized and infected over 150 patients, with most patients being identified at the ICU [15]. Multiple sources and transmission routes have been identified since; with sinks as main source [15, 16]. However, the contribution of each transmission route remains unknown. Therefore, the aim of this study is to quantify the relative importance of each route at the ICU by applying a mathematical transmission model using admission and discharge dates as well as surveillance culture data of patients.

## Methods

### Setting

This retrospective study was conducted at the adult ICU wards of the Erasmus MC in Rotterdam, the Netherlands, using data from January 1st, 2010 until May 18th, 2018. The end date of this period was due to the move to a new hospital. In this 1200-bed university hospital, all medical specialties are available. The adult ICU comprised two high-level ICU wards located on the third and the tenth floor of the adults’ hospital building, and consisted of a total of 34 single-occupancy rooms, of which 7 with anteroom (i.e., isolation rooms). The nurse per patient ratio was between 1:1 and 1:2, the (assistant) doctor per patient ratio was between 1:8 and 1:16, and the intensivist per patient ratio between 1:8 and 1:32 depending on the time of the day. The ratios remained stable over the study period. Nurses worked only on a dedicated ICU ward, whereas doctors may have switched between both ICU wards. However, doctors did not visit the same ward on the same day. In addition, no movement of patients was recorded between the wards (during the same hospital stay) and thus, we treated these ICU wards as separate entities with no transmission between them. At the ICU, patients expected to be on a mechanical ventilator for > 48 h or anticipated to be admitted to the ICU for > 72 h received selective digestive tract decontamination (SDD) as described before, including 4 days of either cefotaxime or ceftriaxone intravenously [17]. During the study period, the SDD regimen did not change, nor did the empirical antibiotic therapy regimen. In our institution, there is an established antimicrobial stewardship program including daily interdisciplinary rounds with intensive care specialists and microbiologists/infectious disease specialists on the ICUs as well as an antibiotic guideline.

General IPC measures were installed after each VIM-PA case was identified (e.g., contact isolation; using gloves and gowns when entering the patient room). However, in 2011 these measures were intensified, and twice-weekly screening for VIM-PA was implemented. An overview of all IPC measures implemented or executed during the study period is available in Additional file 1: Supplement 1.

Written approval to conduct this study was received from the medical ethical research committee from the Erasmus MC (MEC-2015-306). All data were anonymized before analysis.

### Data

We included all admission data and surveillance cultures from two distinct ICU wards in the time period 01/01/2010 till 18/05/2018. As the nurses and doctors remained on one of the two wards during a shift and no movement of patients was recorded between the wards (during the same hospital stay), we treated these ICU wards as separate entities with no transmission between them. If the admission date of a patient preceded the study period, it was set to the beginning of the study period. If the discharge date of a patient lied outside the study period, it was set to the end of the study period. We included all results from throat and rectum cultures that were part of regular VIM-PA and SDD surveillance. Non-surveillance, clinical cultures were excluded to avoid the introduction of selection bias. In particular, clinical cultures are often taken from other sites than screening cultures. This creates a bias as the sensitivity of the cultures may depend on the site, but also because colonization on sites of clinical cultures may not be representative for the other body sites used for screening cultures. Thus, patients with additional clinical cultures may have an increased chance of VIM-PA to be detected. All data were de-identified and anonymized prior to the analysis.

### Microbiology

For the SDD screening, throat and rectum samples were directly cultured on either an ESBL CHROMagar plate (BD diagnostics, Breda, The Netherlands) or CHROMID ESBL (bioMérieux, Marcy l’Etoile, France), a blood agar, and a MacConkey agar (BD diagnostics). A PCR for detection of *bla*_VIM_ was performed on all *P. aeruginosa* isolates with intermediate or resistant susceptibility results for imipenem (MIC ≥ 8 or disk diffusion of < 17 mm) and tobramycin (MIC > 4) as well as isolates with additional “highly-resistant microorganism” profile and imipenem resistance [15]. For the VIM-PA screening, throat and rectum samples were inoculated in Tryptic Soy Broth with ceftazidime (2 mg/L) and vancomycin (50 mg/L). After overnight incubation, an in-house *bla*_VIM_ PCR test was done on the broth [16]. Positive PCR results were confirmed by subculturing the broth on either blood agar or MacConkey agar (BD Diagnostics); *P. aeruginosa* growing on this plate was subjected to *bla*_VIM_ PCR. This PCR-based surveillance was introduced in August 2011. Between April 2011 and August 2011, the VIM-PA screening was performed with the enrichment broth and direct subculturing on MacConkey agar. Prior to 2013, bacterial identification and antibiotic susceptibility testing was performed using VITEK2 (bioMérieux,). After 2013, MALDI-TOF Biotyper (Matrix-assisted laser desorption/ionization-Time of Flight) (Bruker Daltonics, Bremen, Germany) was used for bacterial identification to species level in addition to susceptibility testing with VITEK2. Antibiotic susceptibility results were interpreted according to the European committee on Antimicrobial Susceptibility testing. During the study period, there were no other MBL-positive *Pseudomonas aeruginosa* outbreaks identified. Subsets of VIM-PA isolates were analyzed by one or more genotyping techniques. Results of these analyses are reported in Additional file 1: Supplement 2 and 3.

### Mathematical model

The underlying model is a Susceptible-Infected (SI) model (e.g., [18]). We assumed that all patients admitted to an ICU ward either belong to the susceptible (VIM-PA negative) or colonized (VIM-PA positive) compartment at any given time. The latter includes patients with asymptotic carriage as well as those with a VIM-PA infection. As such, we did not distinguish colonization and infection. In addition, we assumed that every admission is a new patient and once colonized, patients remained colonized with the same level of infectiousness throughout their stay. Events were assumed to occur in daily intervals.

**Fig. 1:**
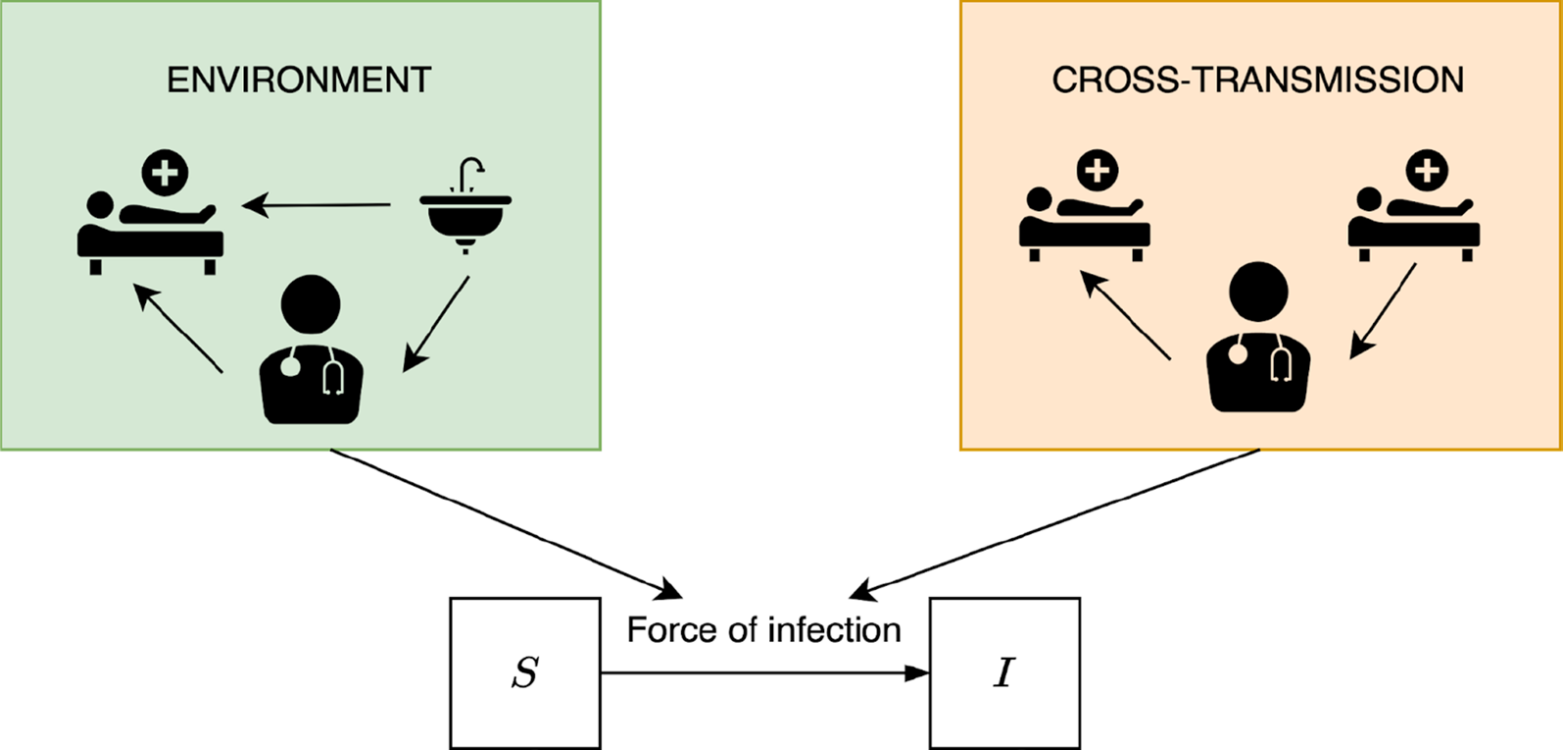
Illustration of the considered transmission routes and the basic transmission model. Patients are assumed to be either susceptible (S) or colonized (I) with VIM-PA. The rate at which susceptible patients may become colonized is represented by the force of infection and dependent on the routes of transmission. Two transmission routes are distinguished: Environmental route (green), mainly caused by transmissions from persistent environmental sources and cross-transmission (orange), i.e., transmissions from other colonized patients. In both routes, HCWs represent vectors of transmission

A susceptible patient may enter the ICU already colonized (with probability $\phi$), or may become colonized at a certain transmission rate $\lambda$. We assumed two different modes of transmission through which colonization can be acquired. The schematic illustration of the model and the transmission routes is given in Fig. 1. Each route induces different patterns in the prevalence time series on the basis of which they may be distinguished statistically. *Cross-transmission*, i.e., colonization caused by (direct) transmissions from other colonized patients present at the same time on the same ward, is dependent on the fraction of colonized patients in the ward. The probability of colonization due to cross-transmission is therefore proportional to the number of colonized patients present in the ward. This assumption is based on the stable nurse-to-patient ratio observed over the whole study period. Consequently, HCWs can be assumed to have a fixed number of patient contacts per day. Since the mobility of patients in ICUs is usually restricted (due to their health status), cross-transmission typically occurs via temporarily contaminated hands of health-care workers (HCWs). We did not model the population of HCWs explicitly but rather assumed direct patient-to-patient transmission with HCWs representing vectors of transmission. Next to cross-transmission, patients may become colonized at a constant per capita rate $\alpha$. In general, this transmission route may be due to, persistent environmental contamination, or introductions from other parts of the hospital, or rarely long term HCW carriers. For VIM-PA the main sources of this transmission route are persistently contaminated environments, such as sinks. We will, therefore, refer to this route as *environmental route.* The force of infection, i.e., the per capita rate of colonization, is modeled as

1 $$\lambda \left( t \right) = \alpha + \beta \frac{I\left( t \right)}{{N\left( t \right)}}$$

where $I(t)$ is the number of colonized patients, $N\left(t\right)$ is the total number of patients currently present in the ward at time $t$, and $\alpha$ and $\beta$ are the transmission rates for the environmental route and cross-transmission, respectively. Based on these parameters, the proportion of acquired colonizations assigned to each route, i.e., the relative contribution of the transmission routes to the overall number of acquired colonizations can be estimated (e.g., [14]).

### Estimation procedure

In the analysis, we used the day of admission and discharge, and the day and result of surveillance cultures as input data for the model. Patients may be admitted to the ward either uncolonized or already colonized. The probability of the latter is defined as the importation probability $f$. The rate at which a susceptible patient may transition to being colonized is given by Eq. (1). The colonization state of a patient is determined by the surveillance cultures provided to the model. Since these culture results are typically intermittent and imperfect, we allow false negative results and colonization results to be imputed in our model. The latter is implemented as a “data-augmentation” step where the surveillance data is augmented for patients with missing or imperfect colonization results. A positive colonization result is added, moved or removed depending on the likelihood (the joint probability of the observed data given the estimated parameters). We define the test sensitivity $\phi$, i.e., the probability that a colonized patient has a positive result.

Patients that were readmitted to the ICU (even when transferred from another ward) were treated as new admissions and their colonization status depends on the results of the (new) surveillance cultures.

We estimated the transmission parameters, the relative contribution of the corresponding transmission routes as well as the importation probability and test sensitivity based on a Bayesian framework using a data-augmented Markov chain Monte Carlo (MCMC) simulation method [11]. The parameters are estimated by fitting the stochastic transmission model to observed data. The main idea is to fit the prevalence pattern resulting from the model to the observed timeseries patterns of the prevalence.

### Implementation

The MCMC algorithm was run for 1,000,000 iterations. A thinning factor of 10 and a burn-in of 30,000 iterations were used. In each iteration, 20 data-augmentation steps were performed with each augmentation chosen at random. The MCMC algorithm was implemented in C++ and the analysis of the output was performed in R (Version 4.0.1) [19]. The data and code are publicly available from: https://github.com/tm-pham/transmission_routes_erasmusMC.

## Results

### Descriptive data analysis

**Fig. 2:**
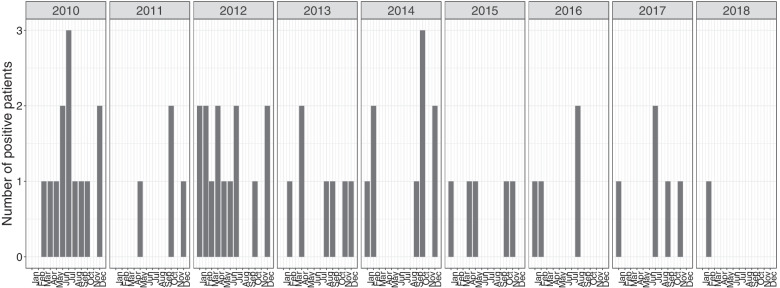
Number of VIM-PA positive patients in the ICU for adults at Erasmus MC, 2010–2018. The date of first positive culture was used. Data for the two ICUs were combined

An overview of the data used in the analysis can be found in Table 1. Since the two considered ICU wards do not differ from each other in terms of admitted patients (i.e., patients were allocated randomly to one of the two ICU wards), we used a combined data set comprising data of both ICU wards for the estimation. The ICU wards were treated as distinct wards with no transmission between them. Data was collected over a study period of 3058 days. In total, 8814 patients were included in the analysis. There were 62 patients with at least one positive culture and 7487 patients with only negative cultures. In total, 1265 patients who were admitted to one of the two ICU wards did not have a culture result. The overall median length of stay was 3.0 days. Patients with an observed colonization had a median length of stay of 13.0 days whereas patients that only had negative culture results had a median length of stay of 3.0 days. The number of patients with positive cultures over time are shown in Fig. 2

**Table 1:** Descriptive statistics for VIM-PA colonization data collected at Erasmus MC, 2010–2018

	No./median (IQR/percentage %)
Study period, days	3058
Admissions	10,408
Number of included patients	8814
Number of patients with readmissions	1128 (12.8%)
Observed number of patients with positive culture(s) for VIM-PA	62 (0.7%)
Number of patients with only negative cultures for VIM-PA	7487 (84.9%)
Number of patients with no cultures	1265 (14.4%)
Length of stay, days	3.0 (2.0–7.0)
* Observed colonized patients*	13.0 (5.0–31.0)
* Observed uncolonized patients*	3.0 (2.0–7.0)
Number of cultures* per included patient	6.0 (4.0–15.0)
Number of combined^†^ cultures per included patient	2.0 (1.0–4.0)
Number of combined cultures per admission	2.0 (1.0–3.0)

VIM-PA, Verona Integron-encoded Metallo-beta-lactamase (VIM)-producing *Pseudomonas aeruginosa*; IQR, interquartile range

*Throat and rectum samples counted individually

^†^Results of throat and recturm samples are combined. If both are negative, the combined culture result is negative. If one is positive, the combined culture result it positive

### Inference results

**Fig. 3:**
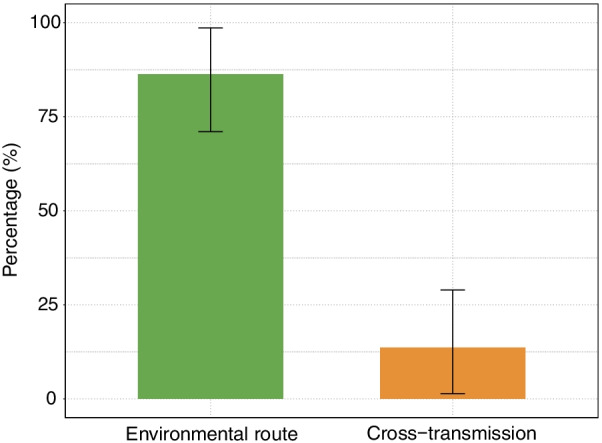
Estimated relative contributions of transmission routes. The height of the bar shows the mean value, the error bars represent the corresponding 95% credibility intervals for the relative contributions of the transmission routes

The estimated parameters are reported in Table 2. We estimated that the majority of the VIM-PA colonizations occurred as acquisitions on the wards and that the majority of these transmissions were due to persistent environmental contamination. In particular, of the estimated 58 (95% credibility interval: 45, 72) acquisitions, approximately 50 acquisitions (86.3%, 95% credibility interval: 71%, 98.6%) occurred via this route leaving 8 (13.7%, 95% credibility interval: 1.4%, 29%) acquisitions due to cross-transmission (see also Fig. 3).

**Table 2:** Summary statistics of the estimated parameters

Parameter	Symbol	Mean (95% credibility interval)
Environmental contamination coefficient	*α*	6.4·10^−4^ (4.1·10^−4^, 9.2·10^−4^
Cross-transmission coefficient	*β*	7.1·10^−3^ (5.6·10^−4^, 1.7·10^−2^)
Probability to be colonized on admission (%)	*f*	0.3 (0.2, 0.5)
Fraction colonized (%)		1.7 (1.6, 1.9)
Test sensitivity (%)	*ϕ*	98.8 (95.6, 100)
Number of acquisitions		58 (45, 72)
Number of importations*		32 (24, 41)
*Contributions*		
Environmental route (%)	$R_{\alpha }$	86.3 (71, 98.6)
Cross-transmission (%)	$R_{\beta }$	13.7 (1.4, 29)

*Colonizations prior to admission to the ward

## Discussion

Our results show that the minority of the transmissions that occurred in the two considered ICU wards was due to cross-transmission. By exclusion, most of the transmissions are estimated to have occurred through persistent environmental contamination. To our knowledge, this is the first study that quantifies the relative contributions of different transmission routes for VIM-PA and confirms the assumption expressed in Voor in ‘t holt et al. [15], that persistent sources in the hospital environment were the main cause of VIM-PA colonizations.

VIM-PA colonizations have been linked to environmental reservoirs such as sinks in other ICUs (e.g., [20–23]). Kizny Gordon et al. [24] summarized studies reporting outbreaks with carbapenem-resistant organisms with a link to the hospital water environment in a systematic review. The authors found that such outbreaks usually involved intensive care settings, the majority of these were caused by *P. aeruginosa*, and that drains, sinks, and faucets were most frequently colonized. Focusing specifically on carbapenem-resistant *P. aeruginosa* outbreaks and all reported sources, Voor in ‘t holt et al. [9] also showed an overrepresentation of sinks as reservoirs. While our method is not able to pinpoint to the exact source of colonizations, we were able to show that cross-transmission, and therefore direct transmission from other patients, was an unlikely cause for the majority of transmissions. In fact, we showed that most transmissions were due to sources that caused a constant risk of colonization independent from other colonized patients. HCW themselves may be such risk as was shown by Foca et al. [25] who described three HCWs with persistent carriage of *P. aeruginosa* on their hands. However, this was associated with nail extenders, candida onychomycosis and an active otitis externa [25]. In the Erasmus MC, we cultured the hands of ICU HCW on two moments (Additional file 1: Supplement 1; February 2010 and May 2011). VIM-PA was not detected in any of these. Furthermore, artificial nails and nail extenders are forbidden in our hospital and were also not observed during the culturing of hands. Therefore, long-term HCW carriers are an unlikely cause and transmissions due to (temporarily) contaminated hands of HCWs have to be linked to other colonized patients present in the respective ward. We acknowledge that hand hygiene compliance was probably not constant over the whole study period but was assumed to be constant in our model (represented by constant transmission parameters). As such, we estimated the average contribution of cross-transmission over 9 years and temporal fluctuations are possible but captured in our model.

Visitors may introduce and transmit microorganisms. However, for VIM-PA, we consider this an unlikely cause. VIM-PA *P. aeruginosa* among hospitalized patients is already < 1%, among non-hospitalized persons this would be even rarer [26]. Thus, by exclusion, the majority of transmissions are assumed to have occurred by persistent environmental sources, confirming the likely role of environmental contamination in the transmission process of VIM-PA in ICUs. These results may be used in the investigation for outbreaks. In fact, genotyping revealed that many sinks were found to be persistently contaminated and isolates could be clustered with patient isolates. Furthermore, the hospital admissions of patients belonging to one cluster did not overlap, making a persisting environmental source a likely explanation (Additional file 1: Supplement 3 and [16]).

Our study encompasses several simplifying assumptions. Firstly, we assumed that every new admission is a new patient. Secondly, while we distinguish two different transmission routes, it is possible that other transmission routes exist that are not included in the model. However, as explained above, other routes than the environment, such as persistent colonization of HCWs, are highly unlikely. The environment as an exclusion-per-definition-category includes a broad range of sources including equipment and inventory. Microbial genotyping data of surveillance samples would allow the identification of specific transmission routes and more detailed quantification of the relative contribution of the transmission routes. Thirdly, we assumed that the environmental route affects all patients in the ICU ward equally. In reality, patients located close to an environmental reservoir may have an increased risk of colonization that will also depend on the microbial load present in the reservoir. Fourthly, non-surveillance or clinical cultures were excluded from our analysis to avoid selection bias. While this excludes potential information, this would likely only affect the uncertainty of our estimates as the data-augmented MCMC method we used imputes missing colonization times. Fifth, SDD might have affected the susceptibility of the patients to and detection rate of VIM-P. aeruginosa. Since the inclusion criteria for the study was hospitalization on the ICU for more than 24 h, and the criteria for starting SDD at our institution is hospitalization on the ICU for more than 72 h or being on the ventilator for more than 48 h, there is a big overlap of the study patients and the patients receiving SDD. In principle, the SDD status of a patient could be included in the model as a risk factor. However, as this information is not available in this data set, we were unable to study this and we also do not expect our overall conclusions to change due to high proportion of patients that received SDD. Finally, we did not include risk factors of different patients and assumed that all patients are equally susceptible to colonization. While the model could be extended to account for the simplifications, this would likely only affect the uncertainty of our estimates but not the main results and conclusions regarding the relative contribution of the transmission routes. We, thus, opted for the simpler model to answer our research question.

## Conclusion

In conclusion, using a large longitudinal data set on admission and discharge times as well as surveillance cultures of patients in two ICUs of the Erasmus MC, we were able to quantify the relative importance of cross-transmission and persistent environmental contamination. Our study contributes to the evidence that persistently contaminated environments in hospital wards may be a major cause of VIM-PA outbreaks. To minimize the transmission risk in wards, reservoirs in the environment should be regularly cultured, thoroughly cleaned, and disinfected. In addition, well-designed sinks and taps may minimize the risk of contamination and consequently spill-over from the environment to patients.

## Supplementary Information

**Additional file 1**.** Supplement 1**. Overview of infection prevention and control measures.** Supplement 2**. Genotyping results of patients.** Supplement 3**. overview of environmental screenings.

## Data Availability

The anonymized data and the full code are publicly available from: https://github.com/tm-pham/VIM-PA_transmission_routes_erasmusMC.git. More details can be provided by the corresponding author upon reasonable request.

## Abbreviations

HCW: Healthcare worker
ICU: Intensive care unit
MDR: Multidrug resistance
*P. aeruginosa*: *Pseudomonas aeruginosa*
VIM-PA: Verona integron-encoded metallo-beta-lactamase-producing *Pseudomonas aeruginosa*
