# Supplementary material for: Routes of transmission of VIM-positive Pseudomonas aeruginosa in the adult intensive care unit-analysis of 9 years of surveillance at a university hospital using a mathematical model
Source: Antimicrob Resist Infect Control. 2022 Apr 4;11:55. doi: 10.1186/s13756-022-01095-x (PMC8981946; doi:10.1186/s13756-022-01095-x)
Supplement: Supplementary file 1 — Additional file 1. Supplement 1. Overview of infection prevention and control measures. Supplement 2. Genotyping results of patients. Supplement 3. overview of environmental screenings. [file 13756_2022_1095_MOESM1_ESM.docx]

**Supplement 1.** Overview of infection prevention and control (IPC) measures installed to prevent transmission of Verona Integron-encoded metallo-beta-lactamase (VIM)-producing *Pseudomonas aeruginosa* (VIM-PA) at the 2 included intensive care units (ICU); January 2010 until May 18, 2018.

**Supplementary Table 1A.** ICP measures implemented for a period of time at the ICU.

| **IPC measure** | **Start date (month-year)** | **End date (month-year)** |
| --- | --- | --- |
| General use of re-usable gowns by HCW. | Jan-10 | Sep-11 |
| General use of gloves and gowns by HCW and visitors (preemptive contact isolation) of all patients admitted at the ICUs. | Oct-11 | Mar-12 |
| General use of gloves and gowns by HCW when having physical patient contact (*i.e.,* not when changing an intravenous fluid bag). | Apr-12 | May-18 |
| The patient room of every patient identified with VIM-PA: daily cleaning, and disinfection with 250 ppm chlorine after discharge. | Aug-10 | May-18 |
| Emphasis (communication by the IPC team) on separation of clean materials from dirty sinks. | Feb-11 | May-18 |
| VIM-PA screening (throat and rectum) of patients on admission and at discharge. | Aug-11 | May -18 |
| VIM-PA screening (throat and rectum) of patients during hospitalization (twice weekly). | Aug-11 | Sep-14 |
| VIM-PA screening (throat and rectum) of patients during hospitalization (weekly). | Oct-14 | Dec-14 |
| VIM-PA screening (throat and rectum) of patients during hospitalization (twice weekly). | Jan-15 | May -18 |
| Electronic flagging in the electronic patient fil of all VIM-PA positive patients. | Sep-11 | May -18 |
| Only allowed to use single-use wash gloves at the ICU. | Dec-11 | May -18 |
| Discontinuation usage of tap water at the ICU, only usage of bottled water allowed. | Dec-11 | May -18 |
| Installation of sink drain plugs as physical barriers against splashing to prevent transmission of VIM-PA from drain reservoirs to the surrounding sink environment.  Pre-intervention phase  Intervention phase  Post-intervention phase  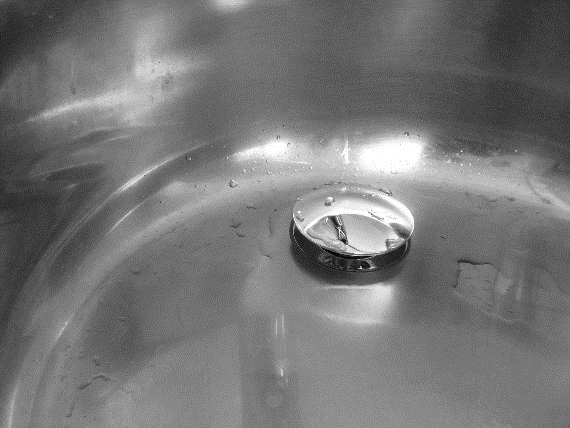 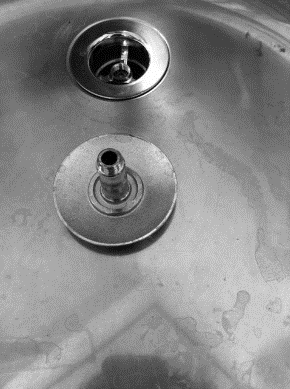 | Jan-13  Jul-13  Aug-13 | Aug-13  Sep-13  Jun-14 |

**Supplementary Table 1B.** Other IPC measures, actions, and meetings at or about the ICU.

| **IPC measure** | **Month-year** |
| --- | --- |
| VIM-PA screening of the ICU environment | Jan-10, Apr-10, Aug-10, Sep-10, Oct-10, May-11, Aug-11, Sep-11, Oct-11, Nov-11, Dec-11, Jan-12, Jan-13, Jun-13, Dec-13, Feb-14, Jun-15, Nov-15, Jan-16, Feb-16, Mar-16, Apr-16, Jul-17, Aug-17 |
| OMT meetings | Sep-10, Jan-11, Feb-11, Mar-11, Apr-11, May-11, Jul-11, Aug-11, Sep-11, Oct-11, Nov-11, Dec-11, Feb-12, Mar-12, Jun-12, Aug-12, Sep-12, Jan-13, Mar-14, Aug-14, Sep-14, Oct-14, Apr-15 |
| VIM-PA screening of hands of healthcare workers (all HCW employed at one ICU) | Feb-10 |
| Sampling of CVVH machines | Feb-10, Aug 12 |
| Replacement of all siphons and drains, including cleaning and disinfection of patient rooms afterwards. | Oct-10 |
| VIM-PA screening (throat and rectum) of all admitted ICU patients | Apr-11 |
| VIM-PA screening of enteral feeding | Aug-11 |
| H_2_O_2_ disinfection of patient rooms of one ICU ward | Aug-11 |
| VIM-PA screening of throat and rectum of all employed HCW at both ICUs | Oct-11, Nov-11 |
| International expert meeting | Dec-11 |
| Regional feedback meeting | Dec-14 |

**Abbreviations**: HCW; healthcare workers, ICU; intensive care unit, VIM-PA; Verona Integron-encoded metallo-beta-lactamase (VIM)-producing *Pseudomonas aeruginosa*, OMT; outbreak management team, CVVH; Continuous Veno-Venous Hemofiltration, ppm; parts per million, IPC; infection prevention and control, H_2_O_2_; hydrogen peroxide.

**Supplement 2.** Genotyping results of the isolates of the 62 patients with positive culture(s) for VIM-PA. Different genotyping methods were used over the study period from January 2010 to May 2018.

1. Diversilab (bioMeriéux)^[[1]](#footnote-1),^^[[2]](#footnote-2)^. *Cluster A, B or other*
2. Multiple Locus Variable number tandem repeat Analysis (MLVA).
   1. Scheme A^[[3]](#footnote-3),^^[[4]](#footnote-4)^
   2. Scheme B^[[5]](#footnote-5)^ *Comparable to Diversilab cluster A, B or unique*
3. Pulsed-Field Gel Electrophoresis (PFGE)^1^. *Comparable to Diversilab cluster A, B or unique.*
4. Whole genome Multi-Locus Sequence Typing (wg-MLST). Performed yes or no at 1) National Institute for Public Health and the Environment (RIVM), Bilthoven, The Netherlands^[[6]](#footnote-6)^, 2) The Erasmus MC University Medical Center, Rotterdam, The Netherlands (Erasmus MC).

| **Patient number** | **1. Diversilab** | **2a. MLVA Scheme A** | **2b. MLVA Scheme B** | **3. PFGE** | **4. wg-MLST available** |
| --- | --- | --- | --- | --- | --- |
| **18** | A | 7 / 382 | B | na | Yes, RIVM |
| **70** | B | 7 / 364 | B | na | na |
| **555** | A | na | na | na | Yes, RIVM |
| **776** | B | na | na | na | na |
| **1015** | Other | na | Unique | na | na |
| **1121** | na | na | na | na | Yes, Erasmus MC |
| **1340** | B | na | na | na | Yes, RIVM |
| **1612** | B | na | na | na | Yes, RIVM |
| **1664** | B | na | na | na | Yes, RIVM |
| **1951** | na | na | na | na | na |
| **2166** | A | na | na | na | Yes, RIVM |
| **2169** | B | na | na | na | Yes, RIVM |
| **2586** | A | 7 / 364 | B | na | na |
| **2754** | B | 7 / 382 | B | na | na |
| **2805** | A | 13 / 68 | A | na | na |
| **2895** | B | 7 / 382 | B | na | Yes, RIVM |
| **2961** | B | na | na | na | na |
| **3079** | B | na | na | na | Yes, RIVM |
| **3789** | B | 7 / 364 | B | na | Yes, RIVM |
| **3838** | B | na | na | na | na |
| **3846** | B | 7 / 382 | B | na | na |
| **3861** | Other | 13 / 68 | A | na | Yes, RIVM |
| **3915** | B | 7 / 382 | B | na | na |
| **4014** | B | na | na | B | na |
| **4087** | B | na | na | na | na |
| **4307** | B | na | na | na | Yes, RIVM |
| **4489** | B | na | na | na | na |
| **4568** | B | 7 / 364 | B | na | na |
| **4720** | B | na | na | na | Yes, RIVM |
| **4826** | B | na | na | na | Yes, RIVM |
| **4868** | B | 7 / 364 | B | na | na |
| **4876** | B | na | na | na | Yes, RIVM |
| **4975** | B | na | na | na | na |
| **5151** | B | na | na | na | Yes, RIVM |
| **5301** | Other | na | na | na | Yes, RIVM |
| **5357** | na | na | B | na | na |
| **6107** | A | na | na | B | na |
| **6612** | Other | na | B | na | na |
| **6873** | Other | na | na | na | Yes, RIVM |
| **6974** | B | na | na | B | Yes, RIVM |
| **7013** | Other | na | na | na | na |
| **7259** | na | na | na | na | na |
| **7374** | A | na | na | na | Yes, RIVM |
| **7771** | Other | na | na | na | Yes, RIVM |
| **8195** | B | na | na | na | na |
| **8317** | B | na | na | na | na |
| **8533** | B | na | na | na | na |
| **8540** | Other | na | na | na | Yes, RIVM |
| **8693** | na | na | na | na | na |
| **9041** | B | 7 / 364 | B | na | na |
| **9218** | B | na | na | na | Yes, RIVM |
| **9471** | B | na | na | B | na |
| **9626** | B | na | na | na | Yes, RIVM |
| **9710** | B | na | B | na | na |
| **9781** | Other | na | na | na | na |
| **9800** | B | na | na | na | Yes, RIVM |
| **10135** | Other | na | na | na | Yes, RIVM |
| **10200** | B | na | na | na | Yes, RIVM |
| **10245** | A | na | na | B | Yes, RIVM |
| **10274** | B | na | na | na | na |
| **10360** | B | na | na | na | Yes, RIVM |
| **10682** | B | na | na | na | Yes, RIVM |

**Abbreviations**: na; not available.

**Supplement 3.** Overview of environmental screenings performed on the two ICU wards for Verona Integron-encoded metallo-beta-lactamase (VIM)-producing *Pseudomonas aeruginosa* (VIM-PA) from January 2010 to May 2018.^[[7]](#footnote-7),^^[[8]](#footnote-8)^

| **year of screening** | **Total number of environmental samples** | **Number of positive samples for VIM-PA (%)** |
| --- | --- | --- |
| total  2010  2011  2012  2013  2014  2015  2016  2017  2018 (Jan – May) | 3489  481  667  308  1629  267  78  25  13  21 | 308 (8.8)  16 (3.3)  19 (2.9)  16 (5.2)  197 (12.1)  28 (10.5)  20 (25.6)  6 (24.0)  4 (30.8)  2 (9.5) |

1. Verfaillie CJ, Bruno MJ, Voor in ‘t holt AF, Buijs JG, Poley JW, Loeve AJ, Severin JA, Abel LF, Smit BJ, de Goeij I, Vos MC. Withdrawal of a novel-design duodenoscope ends outbreak of a VIM-2-producing *Pseudomonas aeruginosa*. Endoscopy. 2015. 47(6), 493-502. PubMed PMID: 25826278. [↑](#footnote-ref-1)
2. Voor in ‘t holt AF, Severin JA, Hagenaars MBH, de Goeij I, Gommers D, Vos MC. VIM-positive *Pseudomonas aeruginosa* in a large tertiary care hospital: matched case-control studies and a network analysis. Antimicrobial Resistance and Infection Control, 2018. 7(32). PubMed PMID: 29492262 [↑](#footnote-ref-2)
3. Van der Bij AK, Van Mansfeld R, Peirano G, Goessens WHF, Severin JA, Pitout JDD, Willems R, Van Westreenen M. First outbreak of VIM-2 metallo-β-lactamase-producing *Pseudomonas aeruginosa* in The Netherlands: microbiology, epidemiology and clinical outcomes. Int J Antimicrob Agents. 2011. 37(6):513-518. PubMed PMID: 21497065. [↑](#footnote-ref-3)
4. Van der Bij AK, Van der Zwan D, Peirano G, Severin JA, Pitout JDD, Van Westreenen, Goessens WHF, MBL-PA Surveillance Study Group. Metallo-β-lactamase-producing *Pseudomonas aeruginosa* in the Netherlands: the nationwide emergence of a single sequence type. Clin Microbiol Infect. 2012. 18(9):E369-E372. PubMed PMID: 22805614. [↑](#footnote-ref-4)
5. Saharman YR, Pelegrin AC, Karuniawati A, Sedono R, Aditianingsih D, Goessens WHF, Klaassen CHW, Van Belkum A, Mirande C, Verbrugh HA, Severin JA. Epidemiology and characterisation of carbapenem-non-susceptible *Pseudomonas aeruginosa* in a large intensive care unit in Jakarta, Indonesia. Int J Antimicrob Agents. 2019. 54(5):655-660. PubMed PMID: 31398483. [↑](#footnote-ref-5)
6. Pirzadian J, Persoon MC, Severin JA, Klaassen CHW, de Greeff SC, Mennen MG, Schoffelen AF, Wielders CCH, Witteveen S, van Santen-Verheuvel M, Schouls LM, Vos MC; Dutch CPE surveillance Study Group. National surveillance pilot study unveils a multicenter, clonal outbreak of VIM-2-producing *Pseudomonas aeruginosa* ST111 in the Netherlands between 2015 and 2017. Sci Rep. 2021. 11(1):21015. PubMed PMID: 34697344. [↑](#footnote-ref-6)
7. SHEA 2020 Decennial (Poster 570). VIM-Positive *Pseudomonas aeruginosa* Sink Colonization Dynamics in Patient Rooms of a Dutch Tertiary Care Hospital. Jannette Pirzadian, Corné H. W. Klaassen, Inge de Goeij, Margreet C. Vos, and Juliëtte A. Severin. [↑](#footnote-ref-7)
8. ECCMID 2019 (poster flash presentation). Limiting spread from environmental reservoirs of VIM-positive *Pseudomonas aeruginosa* in a large tertiary care hospital Jannette Pirzadian, Anne F. Voor in ’t holt, Mehjabeen Hossain, Corné H. W. Klaassen, Inge de Goeij, Lonneke G. M. Bode, Margreet C. Vos, and Juliëtte A. Severin. *Manuscript in preparation*. [↑](#footnote-ref-8)
